# Milk Safety Assessment, Isolation, and Antimicrobial Susceptibility Profile of* Staphylococcus aureus* in Selected Dairy Farms of Mukaturi and Sululta Town, Oromia Region, Ethiopia

**DOI:** 10.1155/2019/3063185

**Published:** 2019-07-28

**Authors:** Sema Regasa, Shimelis Mengistu, Ashebr Abraha

**Affiliations:** ^1^College of Agriculture and Natural Resources, P.O. Box 419, Dilla, Ethiopia; ^2^Haramaya University, College of Veterinary Medicine, P.O. Box 138, Dire Dawa, Ethiopia

## Abstract

A cross-sectional study was conducted to estimate the prevalence of* Staphylococcus aureus* in raw milk and swab, to assess the antimicrobial susceptibility profile, milk handling practice, and its associated risk factors in selected dairy farms of Mukaturi and Sululta Town, Oromia Region, Ethiopia. A total of 247 samples collected from dairy farms were examined using standard microbiological techniques. The antimicrobial susceptibility profiles of the isolates were also investigated. The possible risk factors for* Staphylococcus aureus* contaminations in milk were evaluated through a structured questionnaire. Overall, 16.6% (n= 41) of the samples were positive for* S. aureus*. The prevalence of* S. aureus* was 15.3% from udder milk and 25%, 20%, and 10% from milkers' hand, milking bucket, and drying towel swab, respectively. The prevalence of* Staphylococcus aureus* in milk showed statistically significant variation with respect to age (p≤ 0.001), parity (P≤ 0.001), drainage condition of milking area (P=0.035), study sites (P=0.035), and management system (P=0.035). Majority of the isolates were found resistant to penicillin G (97.6%) and amoxicillin (43.9%). According to this study, 12/28(42.9%)* Staphylococcus aureus* positive raw milk samples had 10^4^-10^5^cfu/ml* S. aureus *count, which is above the recommended level for human consumption. 47.1% of milking persons store milk at room temperature temporarily (between 6 and 12 hrs) till transport to collection center with no means of cooling aid. From consumers 25.6% had no health risk associated with raw milk consumption or aware of milk borne disease associated with drinking raw milk. Thus, 60.5% of milk users had habit of raw milk consumption. The study revealed a prevalence of* S. aureus*, poor milk handling practices, raw milk consumption behavior in study area. Proper handling and hygiene decrease milk contamination by* S. aureus* and make it safe for human consumption.

## 1. Introduction

Milk is considered as nature's single most complete food and is definitely one of the most valuable and regularly consumed foods. But at the same time, it is highly vulnerable to bacterial contamination and hence is easily perishable [1]. Milk and dairy products are considered as the sources of illness associated with milk collection and normal processing conditions that may allow the presence of bacteria in the dairy cows and the dairy environment to be introduced directly into milk. Once introduced, the highly nutritive milk medium supports rapid microbial growth [2].

The safety of raw milk and raw milk products with respect to staphylococcal poisoning is of great concern around the world. Milk can be contaminated by* Staphylococcus aureus* when there is infection of the mammary gland. In addition, it can be contaminated during or after milking by poor hygienic practices, such as improper washing of hands when handling milk storage equipment and coughing or sneezing [3]. In human,* Staphylococcus aureus* is a leading cause of gastroenteritis resulting from the consumption of contaminated food. The most common symptoms are nausea, vomiting, retching, diarrhea, abdominal cramping, and prostration [4].

Higher prevalence of antimicrobial-resistant* S. aureus* was isolated in the dairy farms of highly condensed cows with poor milking hygiene and poor environmental hygiene [5]. Nowadays* S. aureus* strains have developed resistant to the penicillin and to all *β*-lactam drugs. There is no effective long-term decolonization therapy for* S. aureus* carrier [6]. It has been reported that* Staphylococcus aureus* isolates showed multiple resistant to various antimicrobial agents [7].

In Ethiopia, the number of intensive and semi-intensive dairy farms has been increasing from time to time due to urbanization, increased human population, and income growth. However, the management practices of these dairy farms remained traditional [8]. Moreover, in traditional practice the status of cleanliness of the milker, udder the cow, milking environment, and the milking equipment could be the chief source of initial milk contamination and there is lack of standard hygienic condition followed by producers during milk production. Hygienic control of milk and milk products in Ethiopia is not usually conducted on regular bases [9].

Studies conducted in the country showed that* Staphylococcus aureus* is distributed at different parts of Ethiopia and sometimes with higher prevalence. Thus, Fikru (2014) [7] reported 17.2% from farm and abattoir samples at Addis Ababa; Lencho (2015) [10] reported 13.9% from farm samples (milk, udder, hand, and utensil swab) at Ambo and Guder town; and Ayele (2017) [11, 12] reported 19.6% at farm level and 80% at milk collection center at Sebeta. The prevalence of* Staphylococcus aureus* and risk factor contributing to contamination of milk of dairy farm is limited in the study area. Thus, there is a need for study on the status of* S. aureus *and milk handling practice so as to forward the possible management options for* Staphylococcus aureus*. Therefore, the objectives of this study were to estimate the prevalence and antimicrobial susceptibility profile of* Staphylococcus aureus* in milk of dairy cow and swabs from different contact surfaces and assess milk handling practice among selected dairy farms in the study area.

## 2. Materials and Methods

### 2.1. Description of the Study Areas

**Figure 1:**
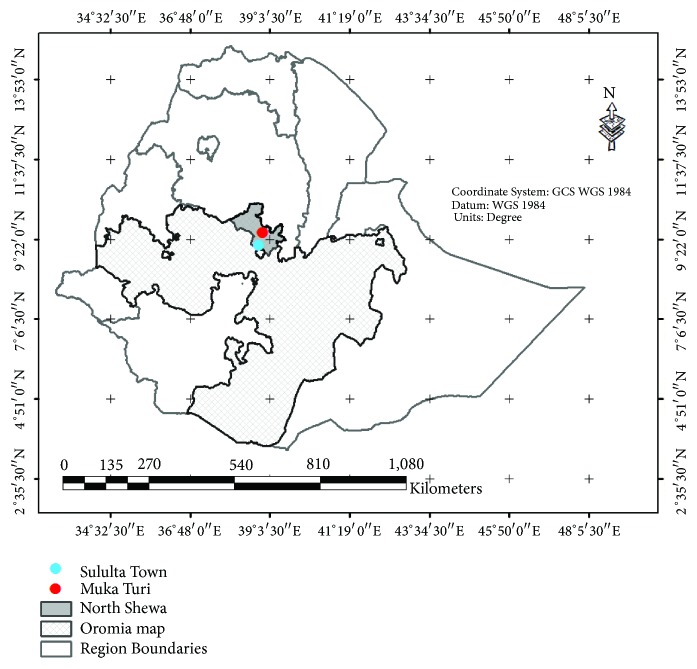
Map of the study area.

The study was conducted in Mukaturi and Sululta town, Oromia National Regional State of Ethiopia as indicated in Figure 1. The study area was selected based on their category of livestock production area, according to the record of dairy development and credit activities.

Mukaturi: Mukaturi is a capital town of Wuchale district located 78 km northwest of Addis Ababa. The district has geographical location of 9°18′-9°46′N and 38°42′-39°07′E latitudes and longitudes, respectively. The agro-climatic zones of the district are temperate (*“Beda”*) ranging from 2300 to 3300, accounting for about 77.2%, subtopical* “Beda-Dare”* ranging from 1500 to 2300 m, accounting for about 20.8%, and tropical* “Gamoji”* ranges from 500 to 1500 m, accounting for about 2% of the district area. The average annual rain fall and temperature of the study area are about 1000 mm and 25°C, respectively. The number of cattle used for milk purpose at North Shoa Zone during 2016 was 236, 808 [11, 12]. The district has a total of 94,141 cattle populations of which 26,142 were cows, 12,193 heifers, and 12,628 female calves and the remaining 43,178 were male cattle [13].

Sululta: Sululta town is one of the towns of Oromia Special Zone surrounding Finfine of Oromia National Regional State. Sululta town is 26 km from Addis Ababa to north and east. The study area is located at 9° 11°N latitude and 38° 45° E longitude. The average elevation in the town is 2765 m above mean sea level. The altitude of the district ranges from 2851 to 3700 meters above sea level. The high annual rain fall is 1447 mm with mean of 1140 mm and minimum of 834 mm. The total cattle population in the district is estimated at 224,600 and 15% are cross-breed [14].

### 2.2. Study Population and Materials

The study population was apparently healthy lactating cows of cross-breed in selected dairy farm which were kept under intensive and semi-intensive management system. Age of the study dairy cows were determined from information of cattle birth records kept, transferred with the cattle as they move from one operation to another or from owner and categorized according to Abera* et al*. (2013) [15] as young (≥ 3 - 5 years), adults (> 6 - ≥ 9 years), and old (> 9 years). Parity was also categorized as few (with 1 - 2 calves), moderate (3 - 4 calves), and many (> 4 calves). Also, lactation stage was classified as early (< 3 months), medium (3 - 6 months), and late (> 6 months). Animal's body condition score was categorized as poor, moderate, and good based on vertebrae at middle of the back, fat deposit behind shoulder and in brisket area, rear view of the hook bone (cross-section), side view of the line between hook and pin bones, and cavity between tail head and pin bone [16]. Drainage conditions of the milking areas were categorized as poor and good from view of accumulated dirty sewage and muddy or properly cleaned area. Milkers who served in dairy farms at selected area were part of the study. In addition to animals, milkers' hands, milking bucket, and drying towels were parts of the study.

### 2.3. Study Design and Sample Type

A cross-sectional study was conducted in the selected dairy farms from November, 2017, to June, 2018, to conduct milk safety assessment and isolation of* Staphylococcus aureus* from udder and different contact surfaces. In addition, antimicrobial susceptibility profile of isolated* Staphylococcus aureus* was performed using standard microbiological methods. Types of samples included were raw milk from cow udder and swab from milkers' hands, milking bucket, and drying towel.

### 2.4. Sample Size Determination and Sampling Strategy

The sample size for this study was determined by the following formula given by Thrusfield (2007) [17]. Therefore, the sample size “n” was calculated as

(1) $$\begin{array}{c} N=\frac{1.96^{2}*Pexp\left(1-Pexp\right)}{d^{2}} \end{array}$$

where n=required sample size, 1.96 = the value of Z at 95% confidence interval, P_*exp*_=expected prevalence, and d =desired absolute precision.

Therefore, the sample size was calculated taking into account 95% confidence interval, desired absolute precision of 5%, and an expected prevalence of 13.9% which was reported by Lencho (2015) [10] from Ambo and Guder Town, which has similar features with the current study area. Accordingly, a total of 183 milk samples were collected by simple random sampling techniques from lactating cows in purposively selected dairy farms. The dairy farms were purposively selected based on the availability of one or more lactating animals and willingness of the dairy farm owners to be part of the study. Then lactating cows from the selected farms were selected using simple random sampling techniques after assigning of identification tags for each lactating animals. Depending on number of workers, frequency of visiting the farm, and materials they used in the farm, 24 swab samples from milkers' hands, 30 swab samples from milking buckets, and 10 swab samples from drying towels were collected. Overall, 247 samples were subjected for microbiological examination.

### 2.5. Sample Collection and Laboratory Analysis

#### 2.5.1. Sample Collection and Transport

According to Tsegalem et al. (2016) [18] twenty-five milliliter volume of raw milk sample was collected aseptically from each 183 apparently healthy lactating cows using sterile universal bottles. The swab samples from milkers' hands, milking buckets, and drying towels were taken using sterile swabs and kept in sample bottles containing sterile physiological saline solution to prevent desiccation. All samples were immediately transported using a box containing an ice to bacteriology laboratory at National Veterinary Institute, DebreZeit, and the samples were kept at 4°C for isolation of the target bacteria within 24 hrs of collection.

#### 2.5.2. Isolation and Identification of Staphylococcus aureus

The bacteriological medium used was prepared according to the manufacturer's recommendations and milk samples were subjected to bacterial culture and identification according to the procedures described by Quinn et al. (2002) [19]. Briefly, a loop full of milk samples and swabs were inoculated on blood agar base enriched with 7% sheep blood and incubated aerobically at 37°C for 24-48hrs. The presence of* Staphylococcus* was confirmed based on colony morphology; Gram's reaction; cellular morphology and organization; and catalase test. Suspected colonies were subcultured on mannitol salt agar and incubated aerobically at 37°C for 24-48hrs. The colonies of staphylococci which produced a yellow pigment on the media were subjected to coagulase tests and cultured on purple base agar (with 1% maltose). Finally,* Staphylococcus aureus *was identified as coagulase-positive and rapidly ferment maltose and change in the medium and colonies appear to be yellow in color [19].

#### 2.5.3. Enumeration of Staphylococcus aureus from Milk Samples

Parallel to inoculation on blood agar, serial dilutions of milk samples were prepared up to 10^−6^ in normal saline water and from each dilution one-milliliter sample suspension was aseptically transferred to Baird Parker as described by Aberra (2010) [20]. The plate containing colonies with typical appearance of circular, smooth, convex, moist, and gray to jet-black, frequently with light-colored (off-white) margin, surrounded by opaque zone and frequently with an outer clear zone in the medium was taken as* S. aureus.* Plates that contained 20-200 colonies were selected for* S. aureus *count [21] and total* S. aureus *colonies from two consecutive plates of each sample were converted into colony forming units per milliliter (cfu/ml) using a formula given by PHE (Public Health England) (2016) [22].

(2) $$\begin{array}{c} N=\frac{\Sigma C}{V\left(n1+0.1n2\right)d} \end{array}$$

where N= number of bacterial colonies counted, C= sum of colonies identified on two consecutive dilution steps, where at least one contained 20 colonies and less than 200 colonies.

V= volume of inoculums on each dish/plate, in milliliter and d= dilution rate corresponding to the first dilution selected (the initial suspension is a dilution).

#### 2.5.4. Antimicrobial Susceptibility Test

The antimicrobial susceptibility profile of* Staphylococcus aureus *isolates was performed using disc diffusion method [23]. The diameters of growth inhibition zone were interpreting and recorded as susceptible, intermediate, and resistant according to the recommendation given by CLSI (2017) [24]. For the susceptibility testing, the following antimicrobial drugs (OXOID, England) and concentrations were used: Amoxicillin (AMX) (25*μ*g), Ampicillin (AM) (10*μ*g), Penicillin (P) (10*μ*g), Tetracycline (TE) (30*μ*g), and Erythromycin (ER) (15*μ*g). Drug selection was based on their accessibility and habitual uses in human and animal medications.

### 2.6. Questionnaire Survey

Structured questionnaire was used to collect information on possible risk factors for* Staphylococcus aureus* contaminations in milk. Risk factors considered in the current study was cleaning conditions of the barn/milking environment, hygiene of milking cows' udder and milk handlers, hygiene of milking equipment with special emphasis on hygiene of milking and milk handling practices, utensils used for milking, milk storage, and uses of milk (for selling or domestic purposes). Furthermore, milk consumption behaviors and their awareness on the risk of zoonotic diseases that are associated with the consumption of raw milk were also assessed.

### 2.7. Data Management and Analysis

The collected data were entered and analyzed using SPSS version 20 computer software. Descriptive statistics were applied to compute prevalence of* Staphylococcus aureus,* percentages of antimicrobial susceptibility profile, and proportions of questioner data. Chi-square test was used to check the presence of association between risk factors and isolation of* Staphylococcus aureus. *The significance level was adjusted at P≤ 0.05.

## 3. Results

### 3.1. Respondents Demography

The study involved 77 respondents who had milk consumer and who had milking personnel intimate to the farm at Mukaturi and Sululta town. From total respondents 43 were milk consumer and 34 were milking personnel. In regard with school education category, of the milk consumers that were interviewed, 58.1% are educated while 52.9% had no formal education from milking personnel. In regard with age category, 60.5% and 47.1% of milk consumers and milking persons, respectively, fall within the age group of 21-30 years. This indicates that the majority of the respondents were in potential productive age (Table 1).

### 3.2. Respondents Knowledge and Practices on Milk Hygiene and Consumption

The study showed that large proportion of milk consumers (48.8%) used milk collection centers as a source of milk. Moreover, most of the milk consumers (72.1%) used plastic container to buy or transport milk. Meanwhile, 48.8% of consumers kept on milk for 6-12 hours under room temperature before consumption while, 37.2% preserve the milk at below 4°C in refrigerator. From respondent information the occurrence of GIT disturbance associated with drinking of raw milk is observed in infant more than children and adults. Also, 25.6% had no health risk associated with raw milk consumption nor are aware of milk borne disease associated with drinking raw milk (Table 2).

As described in Table 3, 76.5% of milking persons practiced udder washing and drying before milking. However, 64.7% did not practice postmilking udder wash and drying and did not use drying towel separately for udder. On the other hand, 58.8% practiced washing of milking equipment and storage container with detergents before milking (Table 3).

### 3.3. Overall Prevalence of Staphylococcus aureus

From the total samples examined, the prevalence of* Staphylococcus aureus* was 16.6%. Based on sample types the higher prevalence of* S. aureus was recorded* in milk samples (15.3), while the lowest was recorded from drying towel (10%) (Table 4).

### 3.4. Risk Factors Associated with Prevalence of Staphylococcus aureus

In the present study, the prevalence of* S. aureus* was found higher in animals of old age, giving many births (>4 calves), and poor body condition than their counter categories. The prevalence of* Staphylococcus aureus *in milk showed statistically significant variation (p≤0.05) with respect to age, parity, management system, and drainage condition of milking area and farming area (Table 5).

### 3.5. Staphylococcus aureus Load in Raw Milk Samples from Cow Udder

The present study revealed that from 28* S. aureus* positive milk samples, 12 (42.9%) had* S. aureus* count which is above the recommended level for human consumption (greater than 20 cfu/ml). From 12* Staphylococcus aureus *positive raw milk samples, nine samples had levels of* Staphylococcus aureus *corresponding to 10^4^cfu/ml and three samples had levels of 10^5^cfu/ml (Table 6).

### 3.6. Antimicrobial Susceptibility Profiles of S. aureus Isolates

The present study demonstrated the existence of alarming levels of susceptibility of* S. aureus* to commonly used antimicrobial agents in the study farms. Thus, 75.6, 56.1, and 51.2% of the* S. aureus* were found to be susceptible to Ampicillin, Amoxicillin, and Tetracycline, respectively. On other hand, 51.2% of* S. aureus *showed intermediate susceptibility to Erythromycin. The resistance profile against Penicillin and Amoxicillin was 97.6 and 43.9%, respectively (Table 7).

Based on analysis of multidrug resistance patterns of* S. aureus* isolates, 2.4% exhibited resistance to Ampicillin, Amoxicillin, Penicillin, and Tetracycline whereas 2.4% isolates were susceptible to all antibiotics used. The most frequent multidrug resistant isolates were those exhibiting resistance to Amoxicillin and Penicillin at a frequency of 21.95%. Meanwhile, 9.76% of isolates showed resistance to Erythromycin, Amoxicillin, and Penicillin (Table 8).

## 4. Discussion

### 4.1. Respondents Knowledge and Practices on Milk Hygiene and Consumption

In the study area, 37.2%, 48.8%, and 14.0% respondents buy milk from farm, collection center, and others sources, respectively. With regard to milk handling, 72.1% of respondents (milk consumers) used plastic containers for milk handling; meanwhile only 37.2% kept milk in refrigeration before consumption. The study also revealed that 60.5% of milk users had habit of raw milk consumption. In agreement with the present findings, study from Sebeta showed that large proportion (66%) of consumers use plastic container and only 10% kept milk in a refrigerator, while 90% of them kept milk at room temperature [11, 12]. Disagreeably, study in Debre-Zeit also reported that 31.8% of dairy producers and 36% consumers had the habit of drinking raw milk [25]. The variation in milk consumption habits could be due to the strong traditional habit of the people in the study area for utilizing raw milk and milk products were greatly at risk of obtaining these pathogen and limit of awareness on milk borne disease.

Dissimilar to this study, [26] in and around Jigjiga City of Somali Region reported that about 92% of respondents did not use udder washing before milking and all the interviewees did not use towel to dry udder after washing. Utensil used for milking and storage determine the safety of milk and milk products. In this study, apart from one all dairy cow milking persons in selected dairy farms practice hand milking and 76.5% used plastic bucket for milking and 29.4% were used for milk storage. Similar to this finding, the works by [26] in and around Jigjiga City of Somali Region reported that all respondents practice hand milking and above 60% of the interviewed householders used plastic jars as milking utensil and transport utensil. The use of plastic and traditional containers can be a potential source for the contamination of milk by bacteria because this allows the multiplication of bacteria on milk contact surfaces during the interval between milking processes. There may be difficulty in removing all milk residues from traditional containers that are porous by nature with the common cleaning systems. In this study, about 55.9% of the respondents clean the barn once per day. In agreement with the report by [26] in and around Jigjiga City of Somali Region about 75% of the respondents clean the barn once per day.

As a result of the current study, from the total of milking persons (47.1%) store milk at room temperature temporarily between 6 and 12 hr till transport to collection centers with no means of cooling aid. This would certainly support the growth and multiplication of* S. aureus* as it is able to survive and multiply in a variety of food substrates, at appropriate temperatures. The overall effect of these poor milk handling practices could lead to contamination of the dairy product as realized in the study.

### 4.2. Overall Prevalence of Staphylococcus aureus

The present study showed that 16.6%* Staphylococcus aureus *isolates were detected out of 247 samples collected: 15.3% originating from raw cows' milk, 25% swabs of milkers' hands, 20% swabs of milking bucket, and 10% swabs of drying towel. The present finding 16.6% is slightly higher when it is compared with a study conducted by [10] who reported 13.9% at Ambo and Guder town. This variation might be due to small number of samples in the current study whereas this finding is in line with reports of [27] at Addis Ababa (15.5%), [7] at Addis Ababa (17.2%), [11, 12] at Sebeta (19.6%), [28] at Addis Ababa (21.13%), [29] at Alage Atvet College Dairy Farm, Ethiopia (21.2%), and [30] from North West India (19.7%). However, the result of the present study showed a slightly lower contamination rate compared to other works[31] who reported 39.1%,* S. aureus* isolates at Asella and [32], who reported 39.09%,* S. aureus* isolates at region of Tirupati, India. In the current study, milk samples were collected directly from cows' udder before contacting milking utensils that might decrease the prevalence of* S. aureus* and this may be attributed to differences in the management practices at farm level. The isolation of* S. aureus* from hands of milkers, milk buckets, and drying towels swabs were 25%, 20%, and 10%, respectively. These clearly indicated that milk handlers, milk buckets, and drying towel, could be the potential sources of contamination of milk with* S. aureus*.

The current finding on the prevalence of* S. aureus* from milkers' hands swabs is in line with the finding of [10], who reported a prevalence of 20% from swabs of milkers' hands in Ambo and Guder town. The prevalence of* S. aureus* in milkers' hands and milking buckets swabs is lower as compared with the report of [11, 12] at proportions of 32% and 11.1%, respectively, at Sebeta. However, [10, 33] reported a far lower prevalence rate (0% and 9%, respectively) of* S. aureus* from milking bucket swab in the country. The dissimilarity of prevalence of* Staphylococcus aureus *isolates probable the hygienic status of equipment sampled of the present study was no good than the previous study. Generally, the occurrence of* S. aureus* in different contact surfaces is supported by the evidence that they are present on the skin of probably 50% or more of healthy individuals as well as in the air, dust, water, and human, and animal wastes [34].

### 4.3. Risk Factors Associated with Prevalence of Staphylococcus aureus

The present study showed significantly high prevalence of* Staphylococcus aureus* in Mukaturi than Sululta and semi-intensive than intensive management system (p=0.035) with prevalence recorded 21.8% in both Mukaturi and semi-intensive management and 10.5% in both Sululta and intensive management system. This is due to association with cows which were maintained in dirty and muddy common barns with bedding materials and failure to use separate towel for individual cows; there could be high chance of contamination of the udder and milk with pathogenic microorganisms.

The present study showed significantly high prevalence of* Staphylococcus aureus* in poor than good drainage condition of milking area (p=0.035) with high prevalence recorded 20.2% in poor and 8.9% in good drainage condition. This is due to association with poor hygiene of milking area; cows which were milking in dirty, muddy, and sewage full drainage area increase milk contamination and favor the proliferation and transmission of* S. aureus* to udder of cow. It was also found that prevalence of* Staphylococcus aureus* increases as parity number increases with statistically significant variation among the categories (P≤ 0.001) with high prevalence recording 5.4%, 15% and 45% in few, mid, and many parity levels, respectively. This result is disagreeable with report of [15] at Adama which has no statistically significant variation. However, it agrees with the findings of [35] in and around Asella town. This could be due to the fact that as the parity number increases there is high degree of contamination of the udder and milk through milking process. In addition to this, large amount of milk is produced and as a result the pressure on the teat canal forces the canals to be opened widely allowing entrance of microbes. The study also revealed that statistically significant association was observed among age categories (p≤ 0.001) with high prevalence recording 10.2%, 11.9%, and 43.5% in young, adult, and old age, respectively. This result agrees with the findings of [35] in and around Asella town and [10] at Ambo and Guder town. This could be due to the fact that old cows are more susceptible to infection than young and adult cows because of weak immune system and they lose their electrolyte by giving large amount of milk production for long time.

The present study showed that the prevalence of* S. aureus* showed that lactation stage has no statistically significant variation (p>0.05). This is in line with the report of [15] at Adama town and [10] at Ambo and Guder town. Additionally, Body scours condition of dairy cow has no statistical association (p>0.05). This is due the fact that* S. aureus* is ubiquitous in nature, with humans and animals as the primary reservoirs. They are present in the nasal passages and throat, in the hair, and on the skin of healthy individuals [6].

### 4.4. Staphylococcus aureus Load in Raw Milk from Cow Udder

The presence of high total* S. aureus* load in raw milk indicates contamination possibly from udder or teat canal of lactating dairy cows. A microbiological study on ready to eat foods in London indicated that total* S. aureus* count (10^2^-10^4^cfu/g/ml) was described as unsatisfactory level of bacterial quality in the foods [36]. According to this study, the total* S. aureus *count in each* Staphylococcus aureus *positive raw milk sample was above 10^4^cfu/ml. Based on the standard level it is unsatisfactory level and milk consumed is a serious risk to the health of the population. The present finding is in line with the findings of [37] that found counts of* Staphylococcus aureus* varying between 10^2^ and 10^5^ cfu/ml in raw milk from* Staphylococcus aureus *positive samples.

### 4.5. Antimicrobial Susceptibility Profiles of S. aureus Isolates

This study presents the susceptibility of* S. aureus* isolates towards Ampicillin, Amoxicillin, and Tetracycline with frequencies of 75.6%, 56.1%, and 51.2%, respectively. However, the isolates were found to be highly resistant to Penicillin G (97.6%). The high resistance pattern of the isolates to penicillin G is relatively similar to the findings reported from the country. Thus, [7, 10–12, 27] reported frequencies of 100%, 98.8%, 96.7%, and 94.6%, respectively. Moreover, the present study showed moderate resistance pattern of* S. aureus *to erythromycin (26.8%) and tetracycline (24.4%). The findings are slightly consistent with the report of [32] in which (13.95%) tetracycline resistance level was seen. However, the current findings are inconsistent with the report of [11, 12] in which (69.1%) erythromycin and (64.7%) tetracycline resistance level was observed. This is might be due to the fact these drugs specifically tetracycline is commonly used in the treatment of infections in the previous study area than the present study area. Lack of stringent regulation and monitoring in the dispensing and use of antimicrobials in the country also might contribute to the occurrence of high antimicrobial resistance to these drugs [11, 12]. Thus, due to the relatively limited access and high price to get the newly developed drugs (like cephalosporin and quinolone) the reports of prevalence of antimicrobial-resistant to relatively low priced and regularly available antibiotics are alarming for a low-income society living in most developing countries, like Ethiopia [38]. In the present study, multidrug resistance pattern of* S. aureus* isolate was reported. Based on analysis of multidrug resistance patterns, 2.4% isolates exhibited resistance to three and four antibiotics with the combination of Ampicillin, Amoxicillin, Penicillin, and Tetracycline while 21.95% isolates showed resistance to the combination of Amoxicillin and Penicillin. On the other hand, 9.76% isolates showed resistance to the combination of Erythromycin, Amoxicillin, and Penicillin. However, 2.4% of isolates were susceptible to all antibiotics used. The probable explanation is,* S. aureus* strains have the capacity to change their resistance behavior to the exposed antimicrobials. The emergence of resistance to many drugs represents public health hazard due to the fact that food borne outbreaks might be difficult to treat and the group of multidrug resistance* S. aureus* in food supply represents a reservoir for communicable resistant genes [38].

## 5. Conclusion

The present study has shown that considerable personnel did not fulfill the standard requirements of milk hygiene at different stages of milk production, storage, and consumption. Thus, majority of the persons use single towel for udder cleaning, did not practice tit dip with antiseptics, and store milk at room temperature for prolonged period of time. In the studied animals, it is evident that large numbers of lactating udders are infected by* S. aureus.* In addition,* S. aureus* is variably occurring on different contact surfaces that have close contact with the milk production process. Age of cow, parity status, farm type, and drainage condition of milking area are important determinates for the occurrence of* S. aureus* in udder of cows. One of the notable findings in the present study was the higher proportion of* S. aureus* contaminated milk samples having a bacterial load above the limit recommended for human consumption. Another notable finding is the occurrence of large proportions of the* S. aureus* isolates resistant to various antimicrobial agents especially to Penicillin and Amoxicillin. Meanwhile, a large proportion of the isolates showed resistance to two or more antimicrobials used, indicating the occurrence of multidrug resistant that may impede effective control of* S. aureus *in udder infection as well as presenting a public health risk due to the spread of drug resistant zoonotic* S. aureus*.

## Figures and Tables

**Table 1:** General information of respondents.

Category	Milk consumer			Milking personnel		
	Mukaturi	Sululta	Total	Mukaturi	Sululta	Total
	(N=24)	(N=19)	(N=43)	(N=16)	(N=18)	(N=34)
*Gender*						
Male	18(75%)	8(42.1%)	26(60.5%)	10(62.5%)	16(88.9%)	26(76.5%)
Female	6(25%)	11(57.9%)	17(39.5%)	6(37.5%)	2(11.1%)	8(23.5%)

*Age*						
18-20	3(12.5%)	2(10.5%)	5(11.6%)	3(18.8%)	8(44.4%)	11(32.4%)
21-30	15(62.5%	11(57.9%)	26(60.5%)	9(56.2%)	7(38.9%)	16(47.1%)
31-40	5(20.8%)	3(15.8%)	8(18.6%)	4(25%)	3(16.7%)	7(20.6%)
41-50	1(4.2%)	3(15.8%)	4(9.3%)	0(0%)	0(0%)	0(0%)

*School*						
Educated	13(54.2%)	12(63.2%)	25(58.1%)	6(37.5%)	10(55.6%)	16(47.1%)
No formal educated	11(45.8%)	7(36.8%)	18(41.9%)	10(62.5%)	8(44.4%)	18(52.9%)

**Table 2:** Information on milk consuming/use and milk handling activity by consumer in the study areas.

Study areas			
*Question items*	Mukaturi (n=24)	Sululta (n=19)	Overall (n=43)

*Milk bought from:*			
Direct from farm	7(29.2%)	9(47.4%)	16(37.2%)
From collection	13(54.2%)	8(42.1%)	21(48.8%)
Other	4(16.7%)	2(10.5%)	6(14.0%)

*Kinds of containers used:*			
Plastic	18(75.0%)	13(68.4%)	31(72.1%)
Stainless steel	6(25.0%)	6(31.6%)	12(27.9%)

*Milk stay at home prior to consumption under room temperature:*			
<2 hours	4(16.7%)	8(42.1%)	12(27.9%)
Between 2-6 hours	2(8.3%)	8(42.1%)	10(23.3%)
Between 6-12 hours	18(75.0%)	3(15.8%)	21(48.8%)

*Where you put the milk at home:*			
With no cooling	17(70.8%)	10(52.6%)	27(62.8%)
In refrigerator	7(29.2%)	9(47.4%)	16(37.2%)

*Habit of milk consumption:*			
Raw	14(58.3%)	12(63.2%)	26(60.5%)
Boiling	10(41.7%)	7(36.8%)	17(39.5%)

*Do you mix fresh and left over milk for consumption:*			
Yes	6(25.0%)	8(42.1%)	14(32.6%)
No	18(75.0%)	11(57.9%)	29(67.4%)

*Do you know any health risk associated with raw milk consumption:*			
Yes	20(83.3%)	12(63.2%)	32(74.4%)
No	4(16.7%)	7(36.8%)	11(25.6%)

*Do you know any GIT disturbance associated with drinking of raw milk:*			
Yes	21(87.5%)	11(57.9%)	32(74.4%)
No	3(12.5%)	8(42.1%)	11(25.6%)

*Which ages have more GIT disturbance associated with drinking of raw milk:*			
Young children	9(37.5%)	5(26.3%)	14(32.6%)
Adult	1(4.2%)	1(5.3%)	2(4.7%)
Infant	14(58.3%)	13(68.4%)	27(62.8%)

*Do you suffer from milk borne infection:*			
Yes	8(33.3%)	6(31.6%)	14(32.6%)
No	16(66.7%)	13(68.4%)	29(67.4%)

n= number of respondents interviewed.

**Table 3:** Hygienic practices during milking and milk handling practice by milking personnel.

Variables	Mukaturi	Sululta	Overall
	(N=16)	(N=18)	(N=34)
*Hand washing before milking*			
yes	10(62.5%)	15(83.3%)	25(73.5%)
no	6(37.5%)	3(16.7%)	9(26.5%)

*Hand washing between milking processes*			
yes	11(68.8%)	18(100%)	29(85.3%)
no	5(31.2%)	0(0%)	5(14.7%)

*Udder washing and dried before milking*			
yes	11(68.8%)	15(83.3%)	26(76.5%)
no	5(31.2%)	3(16.7%)	8(23.5%)

*Udder washing and dried after milking*			
yes	6(37.5%)	6(33.3%)	12(35.3%)
no	10(62.5%)	12(66.7%)	22(64.7%)

*Use drying towel separately for udder *			
yes	5(31.2%)	7(38.9%)	12(35.3%)
no	11(68.8%)	11(61.1%)	22(64.7%)

*Antiseptic use during milking*			
yes	8(50%)	7(38.9%)	15(44.1%)
no	8(50%)	11(61.1%)	19(55.9%)

*Milking utensil used*			
plastic	11(68.8%)	15(83.3%)	26(76.5%)
stainless steel	5(31.2%)	3(16.7%)	8(23.5%)

*Milk storage containers*			
plastic	0(0%)	10(55.6%)	10(29.4%)
stainless steel	16(100%)	8(44.4%)	24(70.6%)

*Detergent use for milk container*			
yes	10(62.5%)	10(55.6%)	20(58.8%)
no	6(37.5%)	8(44.4%)	14(41.2%)

*Barn cleaning*			
Once a day	10(62.5%)	9(50%)	19(55.9%)
Twice a day	6(37.5%)	9(50%)	15(44.1%)

*Milk stored at home under room temperature before sold*			
<2 hours	0(0%)	12(66.7%)	12(35.3%)
Between 2-6 hours	6(37.5%)	0(0%)	6(17.6%)
Between 6-12 hours	10(62.5%)	6(33.3%)	16(47.1%)

N= number of milking personnel involved in the study.

**Table 4:** Percentage of *Staphylococcus aureus* isolate from raw milk and swab samples.

Sample type	Total samples examined	Number of positive samples (%)
Milk sample	183	28 (15.3)
Hand swab	24	6 (25)
Bucket swab	30	6 (20)
Towel swab	10	1 (10)

*Total*	*247*	*41 (16.6)*

**Table 5:** Risk factors for prevalence of *Staphylococcus aureus* in milk.

Risk factors	Total samples examined	No of samples positive (%)	*χ2*	P-value
Area				
Mukaturi	78	17 (21.8)	4.424	0.035
Sululta	105	11 (10.5)		
Age				
Young (≥3-5)	59	6 (10.2)	16.201	≤0.001
Adult (>6- ≥9)	101	12 (11.9)		
Old (>9)	23	10 (43.5)		
Management system				
Intensive	105	11 (10.5)	4.424	0.035
Semi-intensive	78	17 (21.8)		
Parity level				
Few (1-2 calves)	56	3 (5.4)	17.895	≤0.001
Mid (3-4 calves)	107	16 (15)		
Many (>4 calves)	20	9 (45)		
Lactation stage				
Early (<3 moths)	60	8 (13.3)	1.131	0.568
Mid (3-6 moths)	101	15 (14.9)		
Late (>6 moths)	22	5 (22.7)		
Body condition scour				
Poor	7	3 (42.9)	4.286	0.117
Modern	146	21 (14.4)		
Good	30	4 (13.3)		
Drainage condition of milking area:				
Poor	104	21 (20.2)	4.448	0.035
Good	79	7 (8.9)		

**Table 6:** *Staphylococcus aureus* load from raw milk collected directly from udder.

Contaminated raw milk samples	Count of *S. aureus *(cfu/ml)	*S. aureus *log10 cfu/ml
MSD119	1.15 ×10^5^	5.062411
MSD176	5.64 ×10^4^	4.750999
MSD221	7.09 ×10^4^	4.850702
MSD271	5 ×10^4^	4.69897
MSD314	5.27 ×10^4^	4.722035
MIDC24	5.82 ×10^4^	4.764787
MIDC59	5.18 ×10^4^	4.714482
SGF9	7.64 ×10^4^	4.882887
SGtF3	1.06 ×10^5^	5.026793
SGtF4	7.36 ×10^4^	4.867092
SWF6	1.04 ×10^5^	5.015512
SYF2	8×10^4^	4.90309

MSD=Mukaturi Selale Dairy; MIDC=Mukaturi International Dairy Cow; SGF=Sululta Gize Farm; SGtF=Sululta Getinet Farm; SWF=Sululta Wende Farm; SYF=Sululta Yilma Farm.

**Table 7:** Antimicrobial susceptibility test profiles of *S. aureus* isolates (n=41).

Anti-microbial disk	Susceptibility patterns:					
(concentration)	I		R		S	
	No	%	No	%	No	%
AMP (10*μ*g)	4	9.8	6	14.6	31	75.6
ER (15*μ*g)	21	51.2	11	26.8	9	22
AML (25*μ*g)	0	0	18	43.9	23	56.1
P (10*μ*g)	0	0	40	97.6	1	2.4
TE (30*μ*g)	10	24.4	10	24.4	21	51.2

I=intermediate; R=resistant; S=susceptible.

**Table 8:** Multidrug resistance combination of *S. aureus* isolates.

Resistant to drug combination	Antimicrobial	Resistant isolates	
		Number	%
One drug	P	10	24.39
Two drugs	AML,P	9	21.95
	P,TE	2	4.88
	ER,P	5	12.20
Three drugs	ER,P,TE	1	2.44
	AMP,AML,P	1	2.44
	AML,P,TE	3	7.32
	ER,AML,P	4	9.76
	AMP,P,TE	3	7.32
	AMP,ER,P	1	2.44
Four drugs	AMP, AML, P, TE	1	2.44
None	Resistance to none (susceptible to all)	1	2.44
Total		41	100.00

Amoxicillin (AML) (25*μ*g); Ampicillin (AMP) (10*μ*g); Penicillin (P) (10*μ*g); Tetracycline (TE) (30*μ*g); and Erythromycin(ER) (15*μ*g).

## Data Availability

The data used to support the findings can be obtained from the corresponding author upon reasonable request.
